# Interactions between subjective memory complaint and objective cognitive deficit on memory performances

**DOI:** 10.1186/s12877-019-1322-9

**Published:** 2019-10-30

**Authors:** Soowon Park, Ji-Hye Lee, Jiyeon Lee, Youngsung Cho, Hyun Gyu Park, Yongjoon Yoo, Jung-Hae Youn, Seung-Ho Ryu, Jae Yeon Hwang, Jeongsim Kim, Jun-Young Lee

**Affiliations:** 1 Department of Education, Sejong University, Seoul, 05006 Republic of Korea; 2 Gwang Myeong Office Education Wee Center, Gyeonggi-do, 14296 Korea; 3 Department of Psychiatry, SMG-SNU Boramae Medical Center, Seoul National University College of Medicine, Seoul, 07061 Korea; 4 Department of Psychiatry, Bucheon geriatric medical center, Seoul, 14478 Korea; 5 Department of Psychology, University of California Santa Cruz, Santa Cruz, CA 75064 USA; 6 Department of Psychiatry, Seoul National University Hospital, Seoul, 07061 Republic of Korea; 7 Department of Art Therapy & Counseling Psychology, Cha University, Gyeonggi-do, 11160 Korea; 8 Department of Psychiatry, School of Medicine, Konkuk University, Konkuk University Medical Center, Seoul, 05030 Korea; 9 Department of Neuropsychiatry, Hallym University Kangdong Sacred Heart Hospital, 150 Seongan-ro, Gangdong-gu, Seoul, 05355 Korea; 10 Department of Psychiatry and Neuroscience Research Institute, Seoul National University College of Medicine & SMG-SNU Boramae Medical Center, Seoul, 07061 Korea

**Keywords:** Elderly, Cognitive impairment, Subjective memory complaints, Memory, Awareness

## Abstract

**Background:**

Subjective memory complaint (SMCs) is a common trait amongst older population. The subjective cognition about their memory could depend on objective cognition. The aim of the current study was to examine the interaction between subjective memory cognition (i.e., SMC) and objective cognition on cognitive functions in participants from older generation.

**Methods:**

A total of 219 patients, 181 normal control (NC) patients and 38 patients with mild cognitive impairment (MCI), were examined through standardized and comprehensive clinical evaluation and neuropsychological assessment. The Subjective Memory Complaints Questionnaire was used to assess SMCs along with five cognitive tasks were used to evaluate cognitive decline over following areas: verbal memory, visuospatial memory, attention, fluency, and language.

**Results:**

The results of 2 × 2 two-way analysis of variance (ANOVA) showed that there were significant interactions between SMCs and cognitive status (NC, MCI) on memory performances. NC with SMCs showed significantly lower performance in verbal memory and visuospatial memory compared to NCs without SMCs. Conversely, no effect was observed in the MCI group.

**Conclusion:**

There are interactions between subjective cognition (i.e., SMC) and objective cognition (i.e., cognitive status) on memory performances in older adults. The roles of SMCs on memory performances should be interpreted with older adults’ objective cognitive status.

## Background

The aged population is growing worldwide. As the elderly population increases, the prevalence of cognitive disorders, mainly ones related to age such as mild cognitive impairment (MCI) and dementia, are increasing. Decline in Memory is a frequent and common complaint among elderly people, being reported by more than half of the Korean elderly population and causing daily functional impairment during activities [1]. Subjective memory complaints (SMCs) directly impact the elders as they are associated with distress, decline in mental health, and poor quality of life [2].

Petersons suggested a connection between SMCs and MCI, and proposed that the concept for MCI be broadened to include prodromal forms of dementia [3]. Because many studies have focused on SMCs and their relationship with objective cognitive performance, they have become a substantial criteria for diagnosis of MCI, and associated cognitive decline and dementia [4]. Many studies have reported that SMCs predict conversion from normal cognition to dementia amongst older population [5–7]. An autopsy study revealed SMCs were associated with the presence of Alzheimer’s disease (AD) pathology in elderly people, suggesting that SMCs may indicate a level of self-awareness of an ongoing neurodegeneration process [8].

However, studies that examined the relationship between SMCs and objective cognitive functions concluded the absence of relationship between the two elements [9–11]. In the Vienna Transdanube Aging Study, individuals with objective memory impairment often didn’t complain cognitive deficiency, and SMCs were not a reasonable predictor of objective cognitive deficiency amongst the participants [11, 12]. Furthermore, a longitudinal study suggested that cognitive impairments or disabilities with different time progressions were not able to be predicted by using SMCs [13].

Various reasons exist for why studies investigating the association between SMCs and objective cognitive performance are incohesive. First, each study used different neuropsychological tests with different features for different objective cognitive performances. Some studies have used only simple cognitive screening tests for objective memory performance whereas other studies used cognitive tests that measure only specific cognitive functions. Second, the various results may have been affected by cognitive status. A 24-month follow-up cohort study showed that SMCs were linked to a significantly higher risk of dementia in cognitively-intact participants, but not to participants with cognitive impairment [14]. This result indicates that the effect of SMCs on the risk of dementia in elderly population can differ depending on person’s cognitive function.

Moreover, SMCs have been proven to be related to depression and anxiety [15]. Depression has been found to be positively associated with SMCs [16, 17], and memory complaints are more frequent in depressed population [5, 18, 19]. A previous study reported that SMCs may be associated with sub-syndromal depression in cognitively healthy elderly people [20]. In brief, individuals with depressive symptoms may experience a distorted subjective appraisal of their memory. The present study was designed to investigate the role of SMCs by controlling for depression.

## Methods

### Aim

The purpose of this study was to investigate the interactional relationship between subjective and objective cognition on cognitive functions. The effects of interactions between the presence of SMCs and the level of cognitive status (i.e., Normal versus MCI) on cognitive performances were tested.

### Participants

A sample of 219 elder population aged 55 years and older were recruited from Boramae National Hospital, Seoul, South Korea, and a regional dementia clinic for elderly people in Dong Jak district, Seoul, South Korea. Among the 219 patients, 181 were normal controls (NC) and 38 were diagnosed with MCI. Then, the participants were divided into four groups: (A) NC without SMCs, (B) NC with SMCs, (C) MCI without SMCs, (D) MCI with SMCs. We administered a neuropsychological assessment, the Korean version of the Geriatric Depression Scale (GDS) and the Seoul Instrumental Activities of Daily Living (S-IADL). Two or more psychiatrists with expertise in dementia research confirmed the diagnosis through comprehensive neuropsychological examination and the clinical information obtained from the patient and caregiver. A diagnosis of MCI was made according to Petersen criteria [3]; (a) Documented informant perspective memory complaints, (b) Age-related objective memory decline, (c) Preserved general essential cognitive function, (d) Fundamentally integral functional activities, (e) No dementia diagnosed. Exclusion criteria were age under 55 years, illiteracy, lack of education, a history of neuropsychiatric disorders such as dementia, alcohol abuse, head trauma, visual or hearing difficulties, and motor impairment affecting test scores.

### Neuropsychological measures

#### Mini mental state examination

The Mini Mental State Examination (MMSE) is a neurocognitive test with a score ranging from 0 as worst to 30 as best, designed to screen cognitive impairment. This study used the standardized Korean version of MMSE (MMSE-KC), testing on orientation to time and place (10 points), registration (3 points), recall (3 points), attention (5 points), repetition (1 point), language (2 points), and complex commands (6 points) [21].

#### Elderly verbal memory test

The Elderly Verbal Memory Test is designed to scale the measure of verbal memory. In this test, 9 words within 3 categories are provided which are immediately recalled 5 times by the patient. The patient is asked to repeat new sets of nine words immediately after. The patient is then asked to perform a short-term free and cued recall of the initial 9 words with 3 different categories, as well as 20 min delayed free and cued recall with the same initial set of words [22]..

#### Simple Rey figure test

The Simple Rey Figure Test (SRFT) is a simpler version of Rey – Osterrieth Complex Figure Test standardized for elderly patients as the original version included complicated and sophisticated elements which made the test difficult for elders. The SFRT examines constructional strategies, non-verbal memory, visuospatial memory, perception, and motor function. Participants were given geometric figures to copy (copying stage) and later reproduce in delayed free recall (recall stage). The performances in copying and delayed free recall copying were scored [23].

#### Digit span test

The Digit Span Test (DST) assesses participants’ attention and working memory. The test is composed of series of numbers that the participants must read in order the informant has given (forward) and in reverse (backward). The similarity of the pronunciation of certain numbers in Korean was considered when configuring the array of numbers. The forward reading scores assess attention and backward reading scores assess working memory [24].

#### Visual span test

Corsi’s Block Tapping Test (CBTT) represents visuospatial short term working memory, requiring participants to follow the order in which the researcher taps a sequence of multiple (maximum of 10) identical blocks separated in an irregular spatial manner. Similar to the DST, participants must tap the order of the sequence of the researcher forward and then backward. The scores for forward CBTT assess attention and backward CBTT assess working memory [25].

#### Korean Boston naming test – short form

The Korean Boston Naming Test – Short Form (Short K-BNT) is a shorter version of K-BNT, a standardized version of BNT to Korean elderly people, reducing 60 questions to 15 questions with pictures of objects that participants must identify. The questions are composed of pictures of objects familiar and unfamiliar to participants. This test is a visual confrontation naming task that is useful for identifying various types of dementia that cause language disturbance [26].

#### Word fluency test

The Word Fluency Test (WFT), with Controlled Oral Word Association Test (COWAT) as its employed phonetic variant, requires participants to say as many words in a given time (in this case, 60 s) from either a semantic category (e.g., animals or objects) or a phonemic category (e.g., words that start with the letter “a”). For this study, the participants were asked to name as many words as possible related to the semantic category of animals. The scores were measured as the number of words that were appropriate to the given category, excluding repetitions and words unrelated to the given category [27].

### Clinical assessment

#### Subjective memory complaints questionnaire

The Subjective Memory Complaints Questionnaire (SMCQ) is a self-reporting questionnaire for elderly people which includes memory problems in general and daily living. It consists of 14 items reflecting aspects of SMCs, representing metacognition of general and specific memories. Four items assess subjective judgment of memory impairment, and the other 10 items assess memory deficit in everyday life. Higher scores indicate more perceived cognitive decline. Participants with an SMCQ score of 6 and above comprised the SMC group. SMCQ is validated for and adapted to the Korean population [28].

#### Geriatric depression scale – short form

Geriatric Depression Scale (GDS) assess depressive mood in elderly people. We used the shorter version of GDS, standardized for Korean elderly people [29]. Short-form Geriatric Depression Scale (SGDS) is composed of 15 questions with 10 positive and 5 negative questions with yes or no answers. An average score of 8 and above indicates depression [29].

### Seoul instrumental activities of daily living

The Seoul Instrumental Activities of Daily Living (S-IADL) is a reliable and valid tool for the assessment of functional disabilities of Korean dementia patients. S-IADL was developed for evaluating impairment of complex activities in everyday life such as shopping, using transportation, conducting financial affairs, housekeeping, preparing food, using the telephone, or taking medicine. It is composed of 15 questions, and used a 4-point scale. The higher the score, the lower the performance in instrumental daily activities needed for social life [30].

### Procedure

Participants were recruited at Boramae National Hospital and at the regional dementia clinic of Dong Jak district. Participants were 55 years and older and showed no difficulties in performing activities of everyday life. The MMSE-KC, SGDS, and activities of daily living were performed to assess the cognition and depression level of the participants. The neuropsychological assessment was conducted by three psychologists in one-to-one manner in a quiet environment. The details of the study were thoroughly explained to the participants and consents were acquired. The study was approved by the Institutional Review Board of Boramae National Hospital.

### Statistical analysis

The Statistical Package for the Social Sciences 18 (SPSS Inc., Chicago, IL, USA) program analyzed the data. Differences in demographic variables (age, education, gender) and clinical characteristic (SGDS, MMSE) in between-group were verified using either ANOVA or a chi-squared test; Bonferroni adjustments were applied to correct for multiple tests. A 2 × 2 two-way analysis of covariance with age, education, gender and depression as covariates were conducted to assess the interaction of SMCs (presence vs. absence) and cognitive status (NC vs. MCI); Bonferroni adjustments were applied to the post hoc comparisons. The Spearman rank correlation coefficients were used to quantify the relationship between SMCs and cognitive performance across cognitive status groups. Statistical tests were two-tailed, with *p* values of < .05.

## Results

### Demographic characteristics

Demographic and neuropsychological characteristics of the participants are disclosed in Table 1. The average age of the participants was 69.42 years (standard deviation; *SD* = 5.03). No significant difference was observed in age between the NC and MCI groups, but overall, female participants were predominant (M:F = 74:145). Of the 219 participants, 181 had neither particular cognitive impairment nor displayed any objective memory impairment, therefore classified as the NC group. A total of 38 participants were diagnosed with MCI according to Peterson’s criteria [3]. Among the NC group, 116 patients showed neither subjective nor objective memory impairment, whereas 21 patients in the MCI group showed both subjective and objective memory impairment. The mean years of education differed significantly among the groups. Group A (NC without SMCs) had longer education (10.53 years, *SD* = 3.53) than Group D (MCI with SMCs) (7.62 years, *SD* = 3.01). The average MMSE score was higher in Group A (NC without SMCs) than in the other groups (27.91, *SD* = 1.92).

**Table 1:** Demographical and neuropsychiatric characteristics of participants

	Group							Total
	NC		MCI					(*N* = 219)
	(A) No SMCs (*n* = 116)	(B) SMCs (*n* = 65)	(C) No SMCs (*n* = 17)	(D) SMCs (*n* = 21)	*F or x* ^*2*^	*p*	*Post-hoc*	
Age (years)	69.34 (5.05)^a^	68.72 (5.44)	71.65 (3.32)	70.24 (4.43)	1.74	> .1	–	69.42 (5.03)
Education (years)	10.53 (3.53)	9.11 (4.22)	10.00 (3.18)	7.62 (3.01)	4.74	.003	A > D	9.79 (3.78)
Gender (M:F)	39:77	14:51	14:3	7:14	22.29	<.001	–	74:145
GDS	2.37 (2.11)	3.97 (2.21)	2.53 (2.40)	4.76 (2.00)	12.53	<.001	B > A; D > A, C	3.09 (2.32)
MMSE	27.91 (1.92)	26.62 (2.56)	26.00 (2.65)	25.24 (2.32)	12.50	<.001	A > B, C, D	27.12 (2.39)

*Note.* Data are presented as mean (standard deviation) for continuous variables and number (%) for categorical variables

*M* male, *F* female, *GDS* geriatric depression scale, *MMSE* mini-mental state examination, *NC* normal control, *MCI* mild cognitive impairment, *SMCs* subjective memory complaints

### Prevalence of SMCs depending on cognitive status

Of all participants, 39.2% reported experiencing SMCs. In the NC group, 35.9% (65 out of 181) reported SMCs, whereas 55.2% of the MCI group (21 out of 38) reported SMCs. A chi-square test revealed that the percentage of participants that had SMCs differed according to cognitive status; χ2 (1, *N* = 219) = 4.93, *p* = .02). The MCI group seemed more likely to report SMCs than the NC group.

Significant differences were observed in five cognitive subsets, except for the SRFT copying stage, between the NC and MCI groups. However, no significant differences were observed in cognitive performances according to the presence of SMCs even after depression was controlled (Table 2).

**Table 2:** Comparisons of neuropsychological measures between the NC and the MCI group

Measure	NC				MCI				*F* (*df*) 1: Main effect of cognitive status 2: Main effect of SMCs 3: Interaction	*η* ^2^
	(A) No SMCs		(B) SMCs		(C) No SMCs		(D) SMCs		1/2/3	
	*M*	*SD*	*M*	*SD*	*M*	*SD*	*M*	*SD*		
MMSE	27.91	1.92	26.62	2.56	26.00	2.65	25.24	2.32	10.33**/ns/ns	.05/.00/.01
Verbal memory										
Short-term delayed free recall	6.05	1.93	5.43	2.09	2.94	2.38	3.86	2.31	24.81***/ns/ns	.11/.00/.01
Short-term delayed cued recall	6.88	1.67	6.32	1.91	4.35	2.03	5.38	1.77	18.18***/ns/4.45*	.08/.00/.02
Long-term delayed free recall	6.49	1.96	5.78	2.12	3.29	2.62	4.38	2.33	21.52***/ns/3.85^†^	.09/.00/.02
Long-term delayed cued recall	6.96	1.74	6.40	1.70	4.29	2.28	5.24	2.05	20.75***/ns/3.68^†^	.09/.00/.02
Visuospatial Memory										
SRFT copy	15.13	0.89	14.93	1.43	14.97	0.87	14.95	1.05	ns/ns/ns	.00/.00/.00
SRFT immediate	12.84	2.44	11.80	3.78	9.85	3.84	11.21	3.13	6.17*/ns/4.20*	.03/.00/.02
SRFT delayed recall	12.65	2.57	11.19	3.81	8.99	4.24	10.76	4.31	8.02**/ns/6.95**	.04/.00/.03
SRFT recognition	17.41	1.77	17.03	2.26	15.53	1.55	15.90	2.53	10.19**/ns/ns	.05/.00/.00”
Attention										
DST forward span	5.76	1.14	5.62	1.16	5.18	0.95	5.00	0.89	5.49*/ns/ns	.03/.00/.00
DST backward span	4.15	1.07	3.78	1.19	3.47	1.07	3.24	0.77	5.00*/ns/ns	.02/.00/.00
VST forward span	5.49	0.91	5.25	1.06	5.00	0.79	5.00	0.77	4.24*/ns/ns	.02/.00/.01
VST backward span	4.88	1.17	4.32	0.97	4.29	0.92	4.00	1.18	5.02*/ns/ns	.02/.01/.00
Fluency										
Categorical fluency	29.83	5.60	27.51	5.07	25.71	4.97	23.62	4.99	11.35**/ns/ns	.05/.02/.00
Language										
Boston Naming Test	12.13	1.93	11.02	2.18	10.59	2.29	10.38	2.67	5.91*/ns/ns	.03/.01/.01

*Note.* Analysis of covariance was done by using age, education, gender, and geriatric depression scale as covariates

*M* mean, *SD* standard deviation, *NC* normal control, *MCI* mild cognitive impairment, *SMCs* subjective memory complaints, *SRFT* simple rey figure test, *DST* digit span test, *VST* visual span test

^†^*p* < .06; *p < .05; **p < .01; ***p < .001; ns = non-significant

**Fig. 1:**
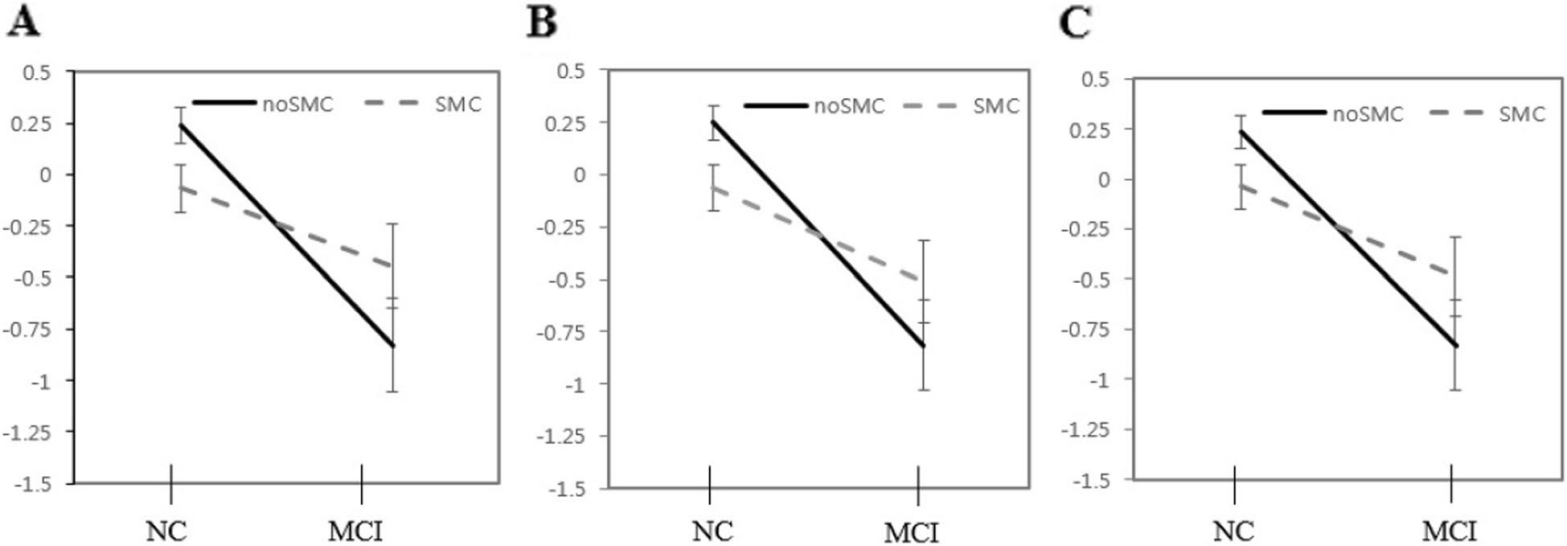
Mean differences of verbal memory performance according to subjective memory complaints (SMCs). **a** Short-term delayed cued recall, **b** Long-term delayed free recall, **c** Long-term delayed cued recall. NC, normal control; MCI, mild cognitive impairment. Values are presented as mean ± SE

**Fig. 2:**
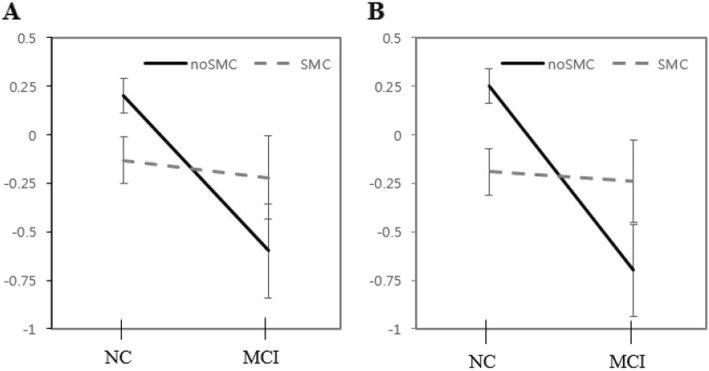
Mean differences of visuospatial memory performance according to subjective memory complaints (SMCs). **a** Simple rey figure test immediate, **b** Simple rey figure test delayed recall. NC, normal control; MCI, mild cognitive impairment. Values are presented as mean ± SE

A significant interaction between the two independent variables (cognitive status, presence of SMCs) was found for tests of verbal memory and visuospatial memory. The interaction between cognitive status and SMCs was significantly related to short-term cued recall of verbal memory and immediate and delayed recall tasks of visuospatial memory (*p* < .05). Furthermore, marginally significant differences were observed in long-term free recall and cued recall tasks of verbal memory (*p* < .06) (Table 2, Figs. 1, 2).

In the interaction between cognitive status and SMCs was observed in the cognitive tasks, NC with SMCs demonstrated significantly lower performance compared to NC without SMCs. Conversely, no effect was observed in the MCI group, which was consistent in every subset of verbal and visuospatial memory (Table 2, Figs. 1, 2).

Table 3 presents the Spearman rank correlation coefficients between SMCs and the neuropsychological measures of the MCI and the NC group. In the MCI group, no significant correlations were found between neuropsychological measures and SMCs. On the other hand, significant and weak association was found between SMCs and the neuropsychological assessments (r_s_ = 0.16–0.25) except for short-term cued recall of verbal memory, SRFT copy, immediate, and recognition, DST forward span, and VST forward span.

**Table 3:** Correlations between subjective memory complaints and cognitive performance across cognitive status groups

	Groups	
	NC	MCI
Cognitive performance		
Verbal memory		
Short-term delayed free recall	−.16^*^	.19^*ns*^
Short-term delayed cued recall	-.14^*ns*^	.31^*ns*^
Long-term delayed free recall	−.18^*^	.22^*ns*^
Long-term delayed cued recall	−.18^*^	.23^*ns*^
Visuospatial Memory		
SRFT copy	.035^*ns*^	.04^*ns*^
SRFT immediate	-.088^*ns*^	.17^*ns*^
SRFT delayed recall	−.17^*^	.24^*ns*^
SRFT recognition	-.044^*ns*^	.18^*ns*^
Attention		
DST forward span	-.08^*ns*^	-.12^*ns*^
DST backward span	−.19^*^	-.11^*ns*^
VST forward span	-.12^*ns*^	.018^*ns*^
VST backward span	−.24^*^	-.11^*ns*^
Fluency		
Categorical fluency	−.18^*^	-.20^*ns*^
Language		
Boston Naming Test	−.25^*^	-.024^*ns*^

Note. Spearman’s correlation analysis was done

*NC* normal control, *MCI* mild cognitive impairment, *SRFT* simple rey figure test, *DST* digit span test, *VST* visual span test

*p < .05 (2-tailed), ns = non-significant

## Discussion

This study aimed to clarify the interaction between cognitive status (i.e., normal control, mild cognitive impairment) and SMCs in order to assess the different role of SMCs across the cognitive status. Neuropsychological measures in verbal and visuospatial memory tasks were particularly lower in cognitively normal participants who reported impairment of subjective memory function, which is inconsistent with MCI. The results of this study indicate that the role of SMCs on memory performance can differentiate depending on the cognitive status.

Previous studies have reported an intricate relationship between SMCs and objective cognitive status. In a study of 302 non-dementia 75-year-olds, approximately 94% of elderly participants with objective memory impairments did not complain about their subjective memory decline. Furthermore, only 6.3% of the elderly participants with objective memory impairments complained about their memory, compared with 10.8% of cognitively healthy participants [11]. The Multifactorial Memory Questionnaire, self-appraised memory and cognitive function in three dimensions in MCI did not correlate with informant-reports and neuropsychological performances [31] . These results showed that the roles of SMCs on memory performances vary depending on the objective cognition.

However, evidence regarding subjective memory loss and cognitive status is inconsistent. Limited screening ability of SMCs for cognitive disorders and deficits was reported by a cross-sectional study [32]. SMCs and informant-reports for cognitive decline significantly discriminated cognitive disorder, including MCI and dementia, from NC. Nonetheless, the informant-reports and collective information of both MCI and dementia demonstrated significantly higher screening accuracy compared to the SMCQ alone, suggesting that combining screening tools can increase the validity of the MCI screening [33].

Even though there are significant correlations between SMCs and cognitive performances in NC, and not in MCI, the correlations were weak. These results indicate that the SMC itself is not a clear criterion for cognitive impairment [34], but researchers should understand SMC can play a role as a moderator in the relationship between cognitive status and objective memory performances. The result showed that the interaction of cognitive status and SMCs was significant only in verbal and visuospatial memory rather than the attention, fluency, and language domains. Because SMCQ is a scale that reflects the function of memory, significant results may be absent in the attention, fluency, or language domains. However, the number of patients in the MCI group was too small to form a decisive conclusion.

SMCs have been reported to be related to depression [15]. Depression, which is positively associated with SMCs, may influence the experience of SMCs [16]. The scores of depression scale were higher in participants reporting SMCs compared to participants who did not report SMCs, which were consistent with other studies [20, 35]. However, SMCs still interacted with objective memory performances after controlling depression in NC and MCI. This result shows that SMCs may not be just a result of depression.

There are several limitations to this study. Firstly, the sample size of the MCI group was relatively small. This may lead to lower statistical power, reducing the chance of detecting a true interaction. Secondly, the present sample consisted of elderly patients who were predominantly female. This limits the generalizability of our findings, however, age, education, gender, and depression were conducted as covariates. It is difficult to exclude the possibility that the elder participants also have high prevalence of comorbidity, which may also have influenced the research. Finally, the study was cross-sectional and was able to explore the cross-sectional interactions but unable to evaluate the ability of the models in predicting conversion to further levels of cognitive disorders. Another limitation of the study is that because the current study could be valid for the Korean population only, further studies are needed to apply it in general.

Nevertheless, this was the first study investigating the effect of subjective memory impairment according to the cognitive status in Korea. Because of the economic burden of treatment and care for dementia, it is crucial to perform appropriate interventions and diagnosis in advance.

## Conclusions

The roles of subjective cognition (i.e., SMCs) on memory performances vary depending on the objective cognition (i.e., cognitive status) which intricately intertwines. SMCs itself are not enough to predict cognitive performances. Researchers and practitioners should simultaneously consider subjective memory complaint and cognitive status of the elderlies to understand their memory performances.

## Data Availability

The datasets used and/or analyzed during the current study are available from the corresponding author on reasonable request.

## Abbreviations

AD: Alzheimer’s disease
ANCOVA: analyses of covariance
ANOVA: analysis of variance
CBTT: Corsi’s Block Tapping Test
COWAT: Controlled Oral Word Association Test
DST: Digit Span Test
EVLT: Elderly Verbal Memory Test
GDS: Geriatric Depression Scale
MCI: mild cognitive impairment
MMSE: Mini Mental State Examination
MMSE-KC: Korean version of Mini-Mental State Examination
NC: normal control
SFRT: Simple Rey Figure Test
SGDS: Short form-Geriatric Depression Scale
Short K-BNT: Korean Boston Naming Test – Short Form
SMCQ: Subjective Memory Complaints Questionnaire
SMCs: Subjective memory complaints
VST: Visual span test
WFT: Word Fluency Test
